# Supplementary material for: A Senescent Cluster in Aged Human Hematopoietic Stem Cell Compartment as Target for Senotherapy
Source: Int J Mol Sci. 2025 Jan 17;26(2):787. doi: 10.3390/ijms26020787 (PMC11766015; doi:10.3390/ijms26020787)
Supplement: Supplementary file 1 [file ijms-26-00787-s001.zip › ijms-3426827-supplementary.pdf]

Supplementary Materials:

Table S1: Clinical Features of the human subjects:

Table S1 shows the clinical features of the initial 12 healthy human subjects from whom we were able to harvest an adequate number of CD34+ cells of high quality for performing scRNA-sequencing studies. For analysis of the rare HSC subclusters, we have recruited additional healthy subjects (2 old and 1 young subjects).

Sample ID	Age	Gender
1	74	F
2	71	M
3	74	F
4	30	F
5	21	F
6	59	M
7	62	F
8	67	M
9	30	F
10	29	M
11	61	M
12	31	M
Additional subjects for HSC subclusters		
13	77	F
14	71	M
15	29	M

Gene sets used for annotation of cell clusters:

We used the following gene sets to define the lineage-specific signatures for the HSPCs (CD34+ cells):
***For hematopoietic lineage development*:**
AVP, CCR7, CD5, CD38, CD79A, CD79B, CEBPA, DNTT, GATA1, GZMH, HBB, MPL, MPO, SPI1, SPIB, VWF

For hematopoietic differentiation pathway:
CD34, AVP, ABI3BP, ADGRG6, ALDH1A1, AREG, BEX1, BST2, CD14, CD44, CD74, CD79A, CD99, CD164, CSF2RB, CSF3R, CXCL8, CRHBP, DDIT3, DUSP1, EIF1, FLT3, FTH1, FUT4, GYPC, HLF, ICAM3, IL7R, IL12A-AS1, ITGA2B, ITGB2, KIT, LAIR1, MEG3, MLLT3, MME, PCDH9, PROM1, SELL, SELP, TM4SF1, THY1

***HSC* –** ADGRG6, CRHBP, HLF, DUSP1, PCDH9
***MLP* –** CD38, GATA3, MME, PRDM1, TAL1, THY-1
***MEP* –** CD38, GATA1, GATA2, GP1BA, GYPA, ITGA2B, TAL1
***GMP* –** CEBPA, CSF3R, SPI1, RUNX1, MPO
***E* –** CA1, HBB, KLF1, TFR2
***G* –** CSF1R, ELANE, MPO, LYZ
***Mega* –** ITGA2B, PLEK, VWF
***Mono* -** ANXA2, LYZ
***DC* –** IGKC, IRF8, SPIB

Additional Gene Sets used in addition to those cited in text and in references (for GSEA):

For Aging signature human HSC:

ATM, CDK6, CHEK1, CHEK2, POT1, SERPINE2, TERF1, ALCAM, ATF3, AXL, BCL2, BCL2L1, BCL6, CCL5, CD9, CDKN1A, CDKN2A, CLU, DUSP1, EGR1, FOSB, FGF7, FTH1, ID1, IGFBP6, IGFBP7, IL1B, IL3RA, IL7, IL9R, IQGAP2, ITGA2, JUN, MAFF,

MIF, MMP9, NAP1L4, NFKB2, NOTCH1, NR4A2, PGF, PLAT, PLK3, PTBP1, PTGER2, PTGES, RELA, SERPINE1, STAT3, SULT1A1, TGFB2, TNFSF10, TP53, TWIST1, VWF

For classification of subclusters in non-primed HSC compartment

CD34, AVP, ABI3BP, ADGRG6, ALDH1A1, AREG, BEX1, CD38, CXCL8, CRHBP, DDIT3, DUSP1, EIF1, FLT3, FTH1, HLF, ICAM3, IL12A-AS1, INSIG1, KIT, MEG3, MLLT3, PCDH9, PROM1, TM4SF1, THY1

Figure S1: Differential expression of aging signature genes in human HSPCs

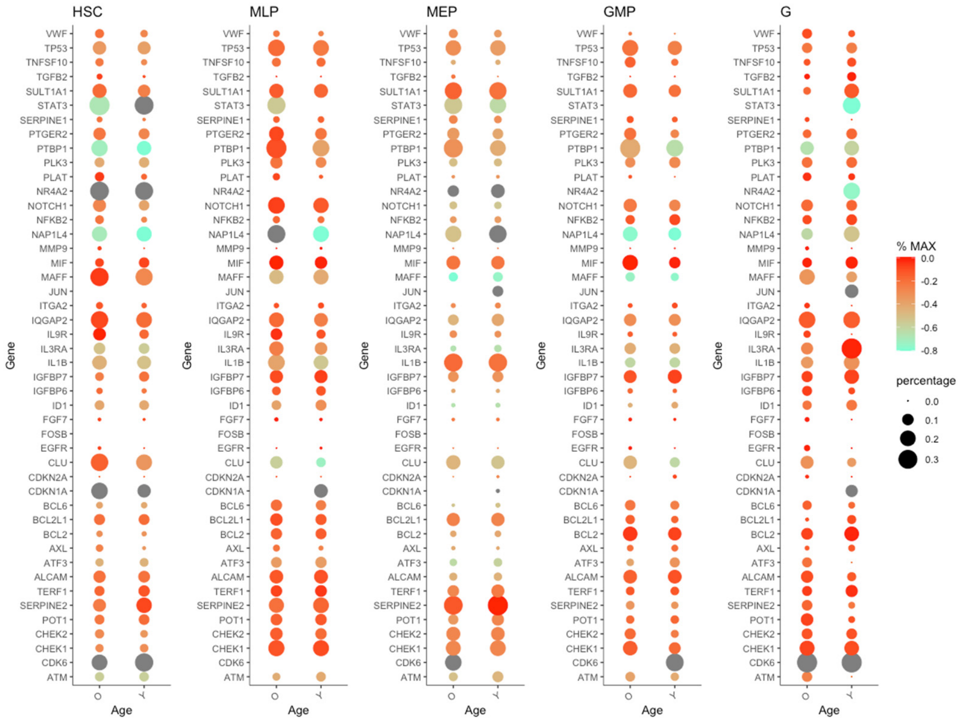

Figure S1 shows the DE of aging signature genes in each of the clusters of HSPCs. The differential gene expressions between old (O) and young (Y) in the respective clusters are shown as dot plots. The relative expression levels of the genes in the gene set "Aging signature human HSC" as listed on the y-axis are represented by the color code of the dots.

Figure S2: Temporal dynamics of CDKN1A expression in developmental trajectory of HSC subclusters

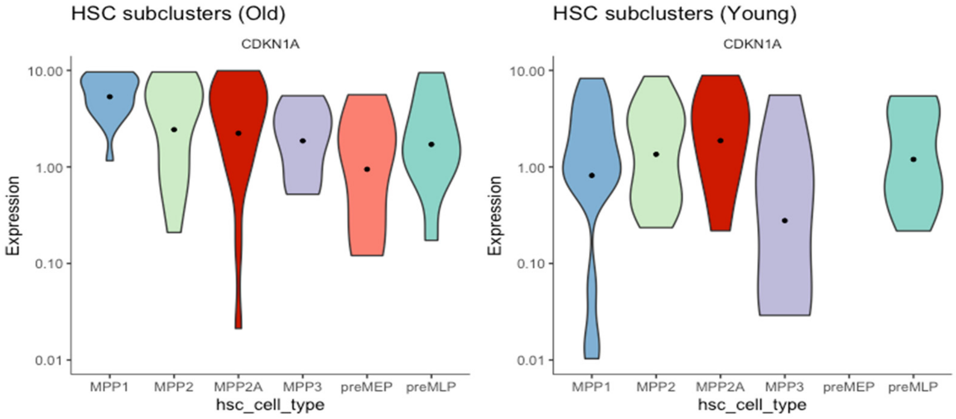

Figure S2 shows that the expression of CDKN1A is significantly increased in the early trajectory of aged HSCs as compared to the corresponding stages in young HSCs. Note that the same color codes for the subclusters are used as in Figures 4A to 4C.

Figure S3: Differential expression of aging signature genes in human HSPCs

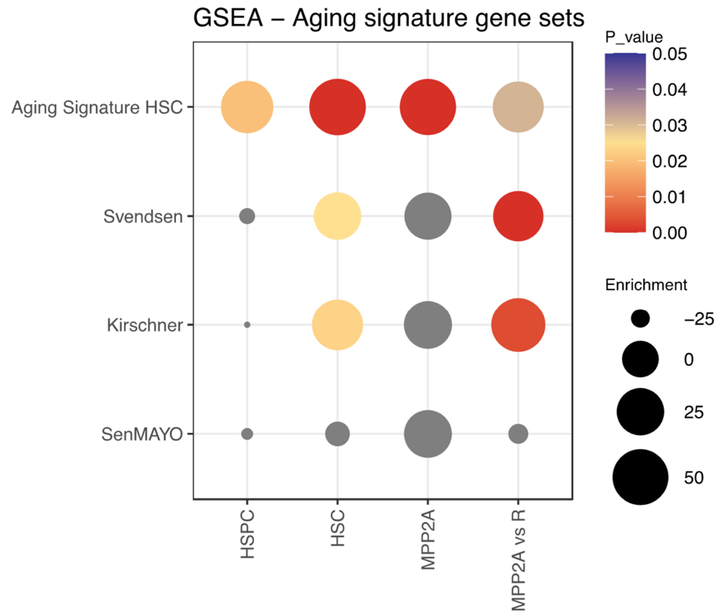

Figure S3 shows the summary of GSEA between the two age groups using the gene sets from "Aging signature human HSC", from Svendsen et al. [67], from Kirschner et al. [37], and from "SenMAYO" [55].
